# Genome-resolved metagenomics analysis provides insights into the ecological role of Thaumarchaeota in the Amazon River and its plume

**DOI:** 10.1186/s12866-020-1698-x

**Published:** 2020-01-15

**Authors:** Otávio H. B. Pinto, Thais F. Silva, Carla S. Vizzotto, Renata H. Santana, Fabyano A. C. Lopes, Bruno S. Silva, Fabiano L. Thompson, Ricardo H. Kruger

**Affiliations:** 1 Department of Enzymology, Institute of Biological Sciences, University of Brasília, Brasilia, 70910-900 Brazil; 2 Department of Civil and Environmental Engineering, University of Brasília, Brasilia, 70910-900 Brazil; 3 Federal Institute of Brasília, Brasilia, 70830-450 Brazil; 4 Laboratory of Microbiology, Federal University of Tocantins, Palmas, 77500-000 Brazil; 5 Department of Genetics, Institute of Biology, Federal University of Rio de Janeiro, Rio de Janeiro, 21941-901 Brazil

**Keywords:** Thaumarchaeota, Amazon River, Amazon River plume, Metagenome-assembled genome

## Abstract

**Background:**

Thaumarchaeota are abundant in the Amazon River, where they are the only ammonia-oxidizing archaea. Despite the importance of Thaumarchaeota, little is known about their physiology, mainly because few isolates are available for study. Therefore, information about Thaumarchaeota was obtained primarily from genomic studies. The aim of this study was to investigate the ecological roles of Thaumarchaeota in the Amazon River and the Amazon River plume.

**Results:**

The archaeal community of the shallow in Amazon River and its plume is dominated by Thaumarchaeota lineages from group 1.1a, which are mainly affiliated to *Candidatus* Nitrosotenuis uzonensis, members of order Nitrosopumilales, *Candidatus* Nitrosoarchaeum, and *Candidatus* Nitrosopelagicus sp. While Thaumarchaeota sequences have decreased their relative abundance in the plume, *Candidatus* Nitrosopelagicus has increased. One genome was recovered from metagenomic data of the Amazon River (ThauR71 [1.05 Mpb]), and two from metagenomic data of the Amazon River plume (ThauP25 [0.94 Mpb] and ThauP41 [1.26 Mpb]). Phylogenetic analysis placed all three Amazon genome bins in Thaumarchaeota Group 1.1a. The annotation revealed that most genes are assigned to the COG subcategory coenzyme transport and metabolism. All three genomes contain genes involved in the hydroxypropionate/hydroxybutyrate cycle, glycolysis, tricarboxylic acid cycle, oxidative phosphorylation. However, ammonia-monooxygenase genes were detected only in ThauP41 and ThauR71. Glycoside hydrolases and auxiliary activities genes were detected only in ThauP25.

**Conclusions:**

Our data indicate that Amazon River is a source of Thaumarchaeota, where these organisms are important for primary production, vitamin production, and nitrification.

## Background

Thaumarchaeota was proposed as an archaeal phylum in 2008 [1]. These organisms were previously known as “mesophilic Crenarchaeota” based on phylogenetic analyses of the large-subunit and small-subunit rRNA gene, which weakly suggested that they form a sister group with Crenarchaeota [1–3]. However, analysis of ribosomal proteins and comparisons of specific proteins showed that Thaumarchaeota represents a phylum more closely related to Euryarchaeota than to Crenarchaeota [1, 4–6]. In addition, genomic analysis showed that Thaumarchaeota possesses genomic features that are not present in either Euryarchaeota or Crenarchaeota [1, 7].

Thaumarchaeota has several lineages that are not yet well defined; the most commonly used nomenclature for these lineages is Groups 1.1a, 1.1a-associated, 1.1b, ThAOA, 1.1c, and 1.3 [8–11]. Groups 1.1a, 1.1a-associated, ThAOA, and 1.1b, which comprise the class Nitrososphaeria, are the only ammonia-oxidizing archaea (AOA) [9, 12, 13]. The gene encoding ammonia monooxygenase subunit A (*amoA*) is used as a marker to study the diversity and abundance of AOA in various habitats [13–25]. However, the lack of cultured representatives of AOA has impeded our study of their physiology. Currently, just ten members of group 1.1a [26–28], three of 1.1b [28, 29], and two from the order Nitrosocaldales [28] could be cultured as pure isolates. Other members have been studied using enrichment cultures [9, 13]; however, Groups 1.1c and 1.3 have no cultured representatives [11, 30]. Thus, most of our information about Thaumarchaeota originates from genomic studies.

Metagenomics studies indicate that Thaumarchaeota is one of the most abundant archaea on the planet [2, 31]. These ubiquitous organisms [9, 14, 32] are present in the euphotic zone and suboxic, eutrophic, oligotrophic, thermophilic, alkaline, and extremely acidic environments [11, 30, 33–38]. They play a major role in nitrification [16, 21, 39, 40] and are crucial in the open ocean, where ammonia concentrations are very low, and ammonia-oxidizing bacteria show low activity [41]. Thaumarchaeota are typically chemolithoautotrophic and fix CO_2_ using a variant of the hydroxypropionate/hydroxybutyrate (HP/HB) cycle, which is the most energy-efficient aerobic CO_2_ fixation pathway [30, 42, 43]. However, despite the importance of Thaumarchaeota, their role in several habitats remains underexplored.

Previous studies have confirmed the presence of Thaumarchaeota in the Amazon River [44, 45], which is considered the world’s largest riverine system, accounting for 20% of the global freshwater discharge into the Atlantic Ocean [46, 47]. The Amazon River and its plume carry a large amount of suspended and dissolved terrestrial materials, such as organic matter and nutrients [48, 49]. The plume can travel many hundreds of kilometers and cover ~ 2 million km^2^, thus contributing to nutrient cycling at a global scale [50–53]. It therefore affects the entire ecosystem, decreasing luminosity and salinity and increasing biological productivity [54]. The high microbial activity in the Amazon River may contribute to its global-scale environmental effects. Therefore, in this study we performed a detailed phylogenetic and functional analysis of three distinct thaumarchaeal genomes obtained from random shotgun sequencing of DNA from the Amazon River and its plume to infer the ecological roles and possible impacts of Thaumarchaeota in these habitats.

## Results

**Fig. 1:**
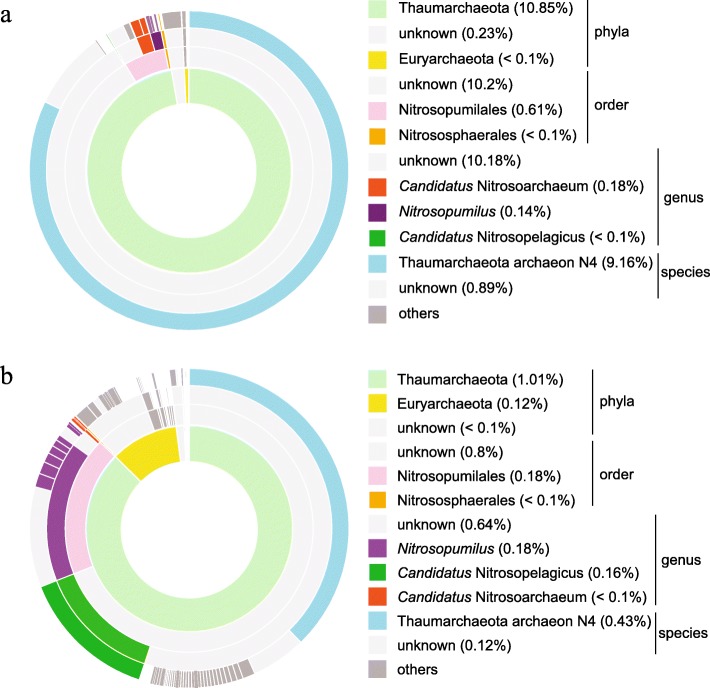
Taxonomic visualization of the archaeal communities in samples from the (**a**) Amazon River and (**b**) Amazon River plume. The outer to inner circles correspond to species, genus, order, and Archaea phyla, respectively. Percentages indicate the relative abundances of these taxa within the entire microbial community

In total, 26,207,858 Paired-end sequencing (PE) reads and 31,379 contigs were recovered from the co-assembly date of Amazon River, plus 38,598,192 PE reads and 138,265 contigs from its plume. Metagenomic analysis showed that Thaumarchaeota was the most abundant archaeal phylum in both the Amazon River and Amazon River plume samples Fig. 1, although the relative abundance of Thaumarchaeota was higher in the river than in its plume. In the Amazon River samples Fig. 1a, Thaumarchaeota comprised approximately 10.85% of the entire microbial while Bacteria 32.91%. The archaeal community is dominated by Thaumarchaeota 97.2%, followed by Euryarchaeota and Crenarchaeota, which comprise less than 1% of the microbial community. The Thaumarchaeota sequences were most phylogenetic related to *Candidatus* Nitrosotenuis uzonensis (Thaumarchaeota archaeon N4; GenBank ID NZ_CBTY000000000.1) (82.1%), followed by other members of order Nitrosopumilales (5.5%) and *Candidatus* Nitrosoarchaeum (1.6%).

In the plume samples Fig. 1b, Thaumarchaeota comprised approximately 1.01% of the microbial community while Bacteria 80.64%, but was still the most representative Archaea in these samples (87.8%), followed by Euryarchaeota (10.4%). The Archaeal phyla Bathyarchaeota, Crenarchaeota, and Woesearchaeota each comprised less than 1% of the microbial community. Thaumarchaeota sequences in the plume were more phylogenetic related to *Candidatus* Nitrosotenuis uzonensis (37.4%), *Candidatus* Nitrosopelagicus sp. (13.9%), and other members of order Nitrosopumilales (18.3%) Fig. 1.

### General genomic analyses

**Fig. 2:**
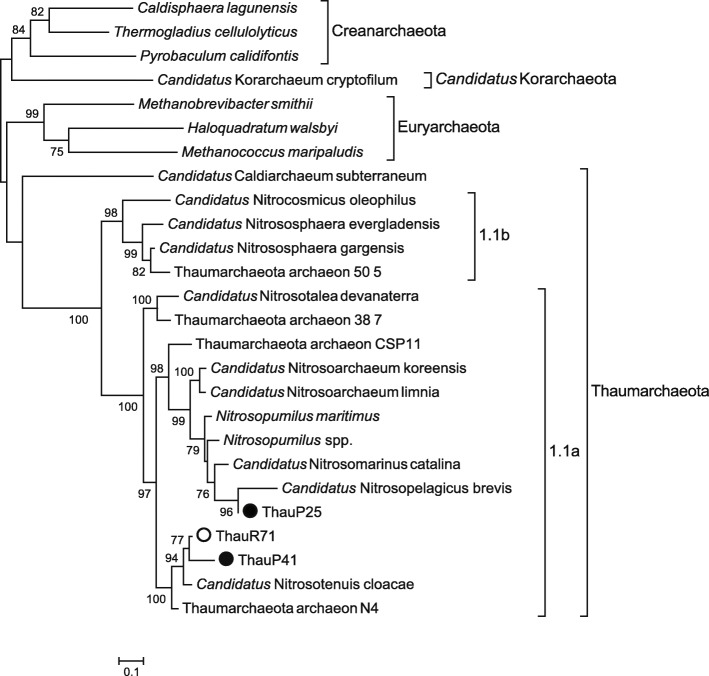
Phylogenetic tree based on six concatenated ribosomal genes. The phylogenetic tree shows the relationship between the Amazon River and plume genomes with other Archaea. Empty circle represent genomes from the Amazon River, and solid circles represent genomes from the Amazon River plume. Sequences were aligned using the multiple sequence alignment program MAFFT, and the phylogenetic tree was constructed using PhyML

One near complete genome was recovered from the co-assembly data of the Amazon River (ThauR71 [1.05 Mbp]), and two from co-assembly data of the Amazon River plume (ThauP25 [0.94 Mbp] and ThauP41 [1.26 Mbp]). Phylogenetic analysis placed all three genomes in Thaumarchaeota Group 1.1a (Nitrosopumilales) Fig. 2. ThauP41 and ThauR71 were placed in the same clade as *Candidatus* Nitrosotenuis cloacae SAT1 and *Candidatus* Nitrosotenuis uzonensis*,* which correspond to the most abundant taxon in these areas. ThauP25 was placed in the same clade as *Candidatus* Nitrosopelagicus, which is more abundant in the Amazon River plume (0.16%) than in the Amazon River (< 0.1%).

The highest ANI value was calculated between ThauP41 and ThauR71 (98.86%), the other values were below to 95%. The ANI of ThauP41 and ThauR71 among *Candidatus* Nitrosotenuis cloacae SAT1 was 76%, whereas between ThauP25 and *Candidatus* Nitrosopelagicus brevis was 81%. The ANI of *Candidatus* Nitrosotenuis uzonensis among the three Thaumarchaeota genomes was below to 73%. All ANI were measured in both directions, but the results never varied by more than 0.01%.

Of the 38 single-copy archaeal genes identified used to measure completeness, the ThauR71 genome contained 37 (97% completeness), the ThauP25 genome contained 35 (92% completeness), and the ThauP41 genome contained 38 (complete), suggesting that each binned genome represented a substantial fraction of a single draft genome. The general features of these three Thaumarchaeota genomes were compared with those of the most closely related genomes: *Candidatus* Nitrosotenuis cloacae SAT1, *Candidatus* Nitrosotenuis uzonensis (Thaumarchaeota archaeon N4), and *Candidatus* Nitrosopelagicus brevis CN25 (Table 1).

**Table 1:** Comparison of general genome features of three Amazon Thaumarchaeota genomes and their phylogenetically closest members

	ThauP25	ThauP41	ThauR71	N4	SAT1	CN25
Genome size, bp	934,797	1,256,699	1,047,532	1,636,125	1,620,156	1,232,128
DNA coding region, bp	878,769	1,159,316	970,632	1,500,929	1,499,274	1,164,105
G + C content, mol%	33.15	37.82	37.99	42.25	41.00	33.16
Total RNA genes, n	47	50	30	46	48	47
tRNA genes, n	44	43	27	41	43	42
rRNA genes, n	0	3	1	3	3	3
Other RNA genes, n	3	4	2	2	2	2
Total number of genes, n	1453	1771	1454	1955	1924	1516
Total CDSs, n (%)	1406 (96.77)	1721 (97.18)	1424 (97.94)	1909 (97.65)	1876 (97.51)	1469 (96.90)
With predicted function, n (%)	920 (63.32)	1119 (63.18)	944 (64.92)	1233 (63.07)	1212 (62.99)	985 (64.97)
Predicted with COG database, n (%)	583 (40.12)	791 (44.66)	660 (45.39)	1006 (51.46)	998 (51.87)	850 (56.07)
Predicted with TIGRFAMs database, n (%)	333 (22.92)	402 (22.70)	343 (23.59)	456 (23.32)	463 (24.06)	413 (27.24)
Encoding signal peptides, n (%)	18 (1.24)	51 (2.88)	36 (2.48)	81 (4.14)	89 (4.63)	40 (2.64)
Encoding transmembrane proteins, n (%)	231 (15.90)	319 (18.01)	255 (17.54)	428 (21.89)	424 (22.04)	289 (19.06)
Without predicted function, n (%)	486 (33.45)	602 (33.99)	480 (33.01)	676 (34.58)	644 (34.51)	484 (31.93)
In internal clusters, n (%)	381 (26.22)	457 (25.80)	234 (16.09)	149 (7.62)	137 (7.12)	66 (4.35)
Metadata						
Isolation source, habitat	Seawater	Seawater	Freshwater	Geothermal hot spring	Wastewater	Seawater
	Atlantic Ocean	Atlantic Ocean	Brazil	Russian	China	Pacific Ocean
Thaumarchaeota group	1.1a	1.1a	1.1a	1.1a	1.1a	1.1a
Species	*Nitrosopelagicus* sp.	*Nitrosotenuis* sp.	*Nitrosotenuis* sp.	*Nitrosotenuis uzonensis*	*Nitrosotenuis cloacae*	*Nitrosopelagicus brevis*

Abbreviations: *CDSs* coding sequences, *COG* clusters of orthologous groups, *rRNA* ribosomal RNA, *tRNA* transfer RNA

The GC content of ThauP41 and ThauR71 (38%), which are both related to *Nitrosotenuis*, was greater than that of ThauP25 (33%), which is more closely related to *Candidatus* Nitrosopelagicus brevis. The GC content of *Candidatus* Nitrosotenuis uzonensis N4 was higher than the genomes obtained in this study (42.25%).

The estimated number of CDSs was 1454 for ThauR71, 1406 for ThauP25, and 1721 for ThauP41, with functions predicted for approximately 63% of these genes (40–45% predicted using the COG database); approximately 33% did not have predicted functions. The percentage of CDSs predicted to be signal peptides was 1.2–2.9% for the three genomes obtained in this study, which is similar to that of *Candidatus* Nitrosotenuis uzonensis, *Candidatus* Nitrosotenuis cloacae, and *Candidatus* Nitrosopelagicus brevis (2.6–4.6%). Similarly, the percentage of CDSs predicted to be transmembrane proteins for the genomes obtained in this study (15.9–18.0%) is similar to that of *Candidatus* Nitrosotenuis uzonensis, *Candidatus* Nitrosotenuis cloacae, and *Candidatus* Nitrosopelagicus brevis (19.0–22.0%).

**Fig. 3:**
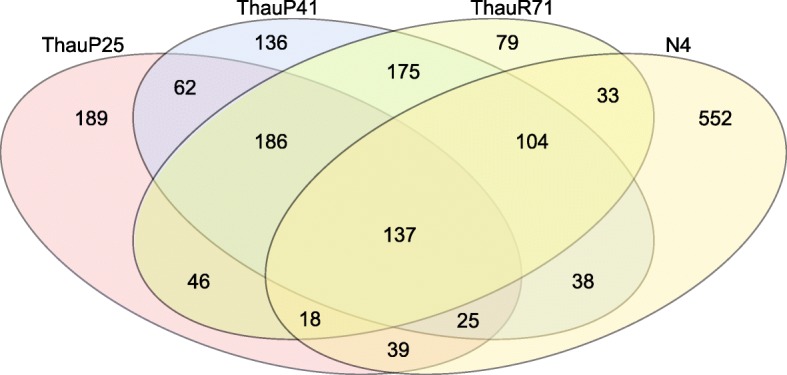
Venn diagram of CDSs that are unique or shared between ThauP25, ThauP41, ThauR71, and *Candidatus* Nitrosotenuis uzonensis N4 (N4)

The distribution of CDSs with predicted function was similar for the Amazon River and plume genomes, which shared 186 CDSs Fig. 3. However, there was a greater number of shared CDSs between ThauP41 and ThauR71 (175) than between ThauP41 and ThauP25 (62), which were from the same habitat. The number of unique CDSs in ThauP25 (189) was higher than that of ThauP41 (136) and ThauR71 (79). *Candidatus* Nitrosotenuis uzonensis N4 shared 39 CDSs with ThauP25, 38 CDSs with ThauP41, and 33 CDSs with ThauP71. A total of 137 CDSs were shared by all four Thaumarchaeota genomes analyzed.

To analyze the three Amazon River and plume genomes, we used the COG database [55] to assign CDSs to the following main functional categories: metabolism, cellular processes and signaling, information storage and processing, and poorly characterized. Within the metabolism category, the highest percentage of genes was assigned to the subcategory coenzyme transport and metabolism (11–12%), which consists of genes involved mainly in the synthesis of cobalamin, biotin, pantothenate, menaquinone, phylloquinone, siroheme, coenzyme F430, coenzyme A, cytochrome c oxidase, coenzyme A, and bacteriochlorophyll c and d. Coenzyme transport and metabolism genes were most abundant in ThauP25 (12.52%), followed by ThauR71 (11.75%). The abundance of genes in other metabolism subcategories was 8–9% for amino acid transport and metabolism, 5–7% for energy production and conversion, 5–6% for nucleotide transport and metabolism, 5% for inorganic ion transport and metabolism, 4–5% for carbohydrate transport and metabolism, 2–3% for lipid transport and metabolism, and 1% for secondary metabolites biosynthesis, transport, and catabolism.

In the cellular processes and signaling category, the highest percentage of genes was assigned to the subcategory posttranslational modification, protein turnover, chaperones (5–6%), followed by signal transduction mechanisms (1–2%), wall/membrane/envelope biogenesis (1–2%), cell cycle control, cell division, chromosome partitioning (1%), defense mechanisms (1%), intracellular trafficking, secretion, and vesicular transport (0.8–1%), and cell motility (0.3–0.8%).

For the information storage and processing category, the highest percentage of genes was assigned to the subcategory translation, ribosomal structure, and biogenesis (15–17%), followed by transcription (4–5%), and replication, recombination, and repair (3%). Genes assigned to the subcategories of chromatin structure and dynamics (< 1%) and RNA processing and modification (< 1%) were not present in all three genomes. Some genes were considered poorly characterized or had only general function predicted (6–7%), or function was unknown (3%) (Table 2). The percentages described above for each category represent the range of these genes in the three genomes obtained in this study.

**Table 2:** Comparison of gene content by COG functional categories

COG		Proteins among:											
		ThauP25		ThauP41		ThauR71		N4		SAT1		CN25	
Code	Description	No.	%	No.	%	No.	%	No.	%	No.	%	No.	%
[C]	Energy production and conversion	51	7.98	52	6.08	41	5.73	67	6.08	69	6.29	60	6.49
[G]	Carbohydrate transport and metabolism	32	5.01	38	4.44	34	4.76	43	3.9	46	4.19	43	4.65
[E]	Amino acid transport and metabolism	60	9.39	72	8.42	65	9.09	102	9.26	106	9.66	90	9.74
[F]	Nucleotide transport and metabolism	37	5.79	56	6.55	46	6.43	60	5.44	59	5.38	53	5.74
[H]	Coenzyme transport and metabolism	80	12.52	97	11.35	84	11.75	101	9.17	102	9.3	100	10.82
[I]	Lipid transport and metabolism	20	3.13	30	3.51	20	2.8	30	2.72	33	3.01	31	3.35
[P]	Inorganic ion transport and metabolism	33	5.16	49	5.73	41	5.73	59	5.35	60	5.47	38	4.11
[Q]	Secondary metabolites biosynthesis, transport and catabolism	6	0.94	13	1.52	13	1.82	14	1.27	16	1.46	13	1.41
[D]	Cell cycle control, cell division, chromosome partitioning	10	1.56	12	1.4	7	0.98	11	1	11	1	8	0.87
[M]	Cell wall/membrane/envelope biogenesis	12	1.88	18	2.11	11	1.54	30	2.72	36	3.28	35	3.79
[N]	Cell motility	5	0.78	3	0.35	2	0.28	20	1.81	20	1.82	5	0.54
[O]	Posttranslational modification, protein turnover, chaperones	40	6.26	50	5.85	49	6.85	56	5.08	64	5.83	46	4.98
[T]	Signal transduction mechanisms	11	1.72	23	2.69	20	2.8	48	4.36	44	4.01	14	1.52
[U]	Intracellular trafficking, secretion, and vesicular transport	9	1.41	7	0.82	6	0.84	12	1.09	10	0.91	9	0.97
[V]	Defense mechanisms	9	1.41	12	1.4	8	1.12	17	1.54	17	1.55	9	0.97
[A]	RNA processing and modification	0	0	1	0.12	1	0.14	1	0.09	1	0.09	0	0
[B]	Chromatin structure and dynamics	1	0.16	2	0.23	2	0.28	2	0.18	2	0.18	2	0.22
[J]	Translation, ribosomal structure and biogenesis	102	15.96	152	17.78	121	16.92	169	15.34	172	15.68	165	17.86
[K]	Transcription	33	5.16	41	4.8	38	5.31	61	5.54	61	5.56	44	4.76
[L]	Replication, recombination and repair	24	3.76	31	3.63	28	3.92	43	3.9	43	3.92	41	4.44
[R]	General function prediction only	41	6.42	67	7.84	55	7.69	106	9.62	88	8.02	79	8.55
[S]	Function unknown	23	3.6	29	3.39	23	3.22	47	4.26	36	3.28	36	3.9
(−)	Not in COG	870	59.88	980	55.34	794	54.61	949	48.54	926	48.13	666	43.93

### Ecophysiology

All three genomes obtained in this study contained genes involved in energy conversion by nitrogen metabolism, oxidative phosphorylation, sulfur metabolism. Genes for carbon fixation were also found in these genomes, they had genes representing a near-complete hydroxypropionate/hydroxybutyrate (HP/HB) cycle and the presence of the gene encoding acetyl/propionyl-CoA carboxylase, which is responsible for HCO_3_ assimilation. However, genes encoding malonyl-CoA reductase and succinate-semialdehyde dehydrogenase were not detected in any of the genomes. The three genomes also contained genes for carbon fixation through the Calvin cycle, but the ribulose 1,5-biphosphate carboxylase were not detected for these genomes.

For nitrogen metabolism, genes encoding ammonia monooxygenase were detected in ThauP41 (*amoA*, *amoB*, and *amoC*) and ThauR71 (*amoB* and *amoC*) but not in ThauP25. All three Amazon River and plume genomes contained *nirK*, which is involved in the production of nitric oxide and nitrous oxide, as well as genes involved in glycolysis, TCA cycle, and oxidative phosphorylation.

Analysis of carbohydrate-active enzymes showed that GH genes were detected just in ThauP25, which have 5 genes from the GH1 family (Additional file 2: Table S1). Genes assigned to the enzyme classes AA (15 genes from AA1 family) were only detected in ThauP25. The families GT1 and GT2 were detected in all three genomes, whereas the ThauP25 has the higher abundance of GT2 (123 vs. mean of 57.5). The family GT66 were just detected for ThauP41 and ThauR71, whereas GT7 and GT90 were just detected for ThauR71. The enzyme classes CE was not detected in any of these genomes.

The genomes ThauP41 and ThauR71 encode a complete assimilatory sulfate reduction pathway, whereas in ThauP25 the *sir* gene was not detected. In addition, genes specific for the anaerobic synthesis of cobalamin were detected in ThauP25, ThauP41 (*gbix*, *cbiG*, and *cbiD*), and ThauR71 (*cbiG* and *cbiD*). Genes of the second cluster of biosynthesis of cobalamin were also detected in ThauR71 (*cobY*, *cobS*, *cobD*, and *cobQ*), the likewise occurs for ThauP25 and ThauP41, though, *cboQ* was not detected.

All three Thaumarchaeota genomes contained the complete set of genes for transport of zinc (*znuA*, *znuB*, *znuC*). Moreover, it was detected genes for transport of phosphate (*pstS*, *pstA*, *pstC*, *pstB*) and lipoprotein (*lolC*, *lolE*, *lolD*) in ThauP41.

## Discussion

Because of the lack of representative Thaumarchaeota genomes, our metagenomics analysis classified many of the sequences obtained in our study as unknown Fig. 1. Most of the sequences of the Thaumarchaeota from the Amazon River and its plume were assigned to members of group 1.1a (Nitrosopumilales) [13] or Marine Group I [2, 19, 56]), suggesting that this is the most abundant archaeal group in these habitats. These results are consistent with previous studies that report that 73% of Thaumarchaeota from estuarine-coastal AOA belong to Nitrosopumilales, and 37% are associated specifically with the clade of *Nitrosopumilus* spp. [13]. The relative abundance of Thaumarchaeota was higher in the Amazon River than in its plume, which might be attributed to the difference in their physical and chemical characteristics. Salinity is lower in the Amazon River (0.01–0.02 g/kg) than in its plume (12.17–36.34 g/kg), as is pH (6.58–6.99 vs. 7.67–8.1) and surface dissolved inorganic carbon (113–502 vs. 818–2030 μmol·C/kg). In contrast, partial pressure of carbon dioxide is higher in the Amazon River (1039–5488 μatm) than its plume (234–569 μatm), as is ammonium (0.72–3.12 vs. 0.0–0.014 μM), nitrate + nitrite (5.64–17.9 vs 0.0–8.52 μM), and dissolved organic carbon (244–381 vs. 61–191 μM) [57]. The higher pressure of carbon dioxide in the Amazon River is probably associated with heterotrophic carbon processing of allochthones organic matter derived from plant material [58, 59]. Ammonium, nitate+nitrite and dissolved carbon concentrations may also be associated with the allochthones organic matter input from de surrounding Amazon forest [60]. Thus, greater availability of nitrogen ammonium, nitrate, and nitrite suggests the pro-dominance of Thaumarchaeota Group 1.1a, since they are nitrifying Archaea. Soil metagenomics studies have also found that Group 1.1a is associated with higher nitrate concentrations and lower pH (5 ≤ pH < 7) [11, 61]. Furthermore, the clades NP-η (*Candidatus* Nitrosotenuis uzonensis) and NP-γ (*Nitrosopumilus* spp.) appear to be associated with low-salinity and freshwater environments [13, 62, 63], which would account for the considerable decrease in Thaumarchaeota in Amazon River plume, since the most abundant taxa in our analysis belong to these clades. Nevertheless, the change from estuarine to marine environment would explain the increase of clade NP-ε (*Candidatus* Nitrosopelagicus), which suggests niche specialization to plume habitat that is correlated with saturation of dissolved oxygen, surface dissolved inorganic carbon, pH, and salinity. Other studies have corroborated the association of this clade with seawater [13]. In addition, clades NP-ε (*Candidatus* Nitrosopelagicus) and NP-γ (*Nitrosopumilus*) are associated with “shallow” water (Water Column A) [13, 64–69]. These findings may account for the abundance of Group 1.1a and the low representation of Groups 1.1a-associated, 1.1b, ThAOA, 1.1c, and 1.3. Thaumarchaeota represent an important fraction of the microbial community in the Amazon River. Although more abundant in the Amazon River, their presence in the plume extending to the Atlantic Ocean.

The genomes recovered in this study (ThauP25, ThauP41, and ThauR71) were assigned to the most abundant taxa of these environments Fig. 1. However, it happens probably because of lack of the known species in the database, since ANI analysis suggests that these genomes represent different Thaumarchaeota species from those of literature. Studies have shown that ANI values equal to approximately 95% correspond to the cut-off values of 70% similarity used on DNA-DNA hybridization as a standard for the classification of new species [70–73]. Therefore, ThauP41, and ThauP25 belong to known genera according to the presented phylogeny.

Our findings suggest that these organisms are prototroph for cobalamin (vitamin B12), biotin (vitamin B7), pantothenate (vitamin B5), menaquinone (vitamin K2), and phylloquinone (vitamin K1) and not require uptake, which has previously been shown by culture of AOA without vitamin supplements [27, 74]. Other studies have reported that Thaumarchaeota plays an important role in the global production of cobalamin [75], thus Thaumarchaeota may be acting and the production of vitamins to this environment.

These three recovered genomes contain genes for the HP/HB cycle, although genes encoding malonyl-CoA reductase and succinate-semialdehyde dehydrogenase were not identified. However, many genes encoding enzymes in Thaumarchaeota do not have homologs in databases and cannot be identified using bioinformatics approach, including a malonyl-CoA reductase in *Nitrosopumilus maritmus* [43, 69, 76]*. N. maritmus* has an alternative enzyme for this step, malonic semialdehyde reductase, which was detected by homology in ThauP25 and ThauP41. AOA are thought to be major contributors to carbon fixation in oceans [77], indicating these organisms contribute to primary production in Atlantic Ocean. On the other hand, the high nutrient and CO_2_ levels in the Amazon River [78] may increase primary production, leading to eutrophication, higher consumption of dissolved oxygen. The presence of genes encoding glycosyl hydrolases detected in ThauP25 and proteins involved in glycolysis and the TCA cycle suggests that these organisms mixotrophic metabolism, which supports the hypothesis that Thaumarchaeota are not only chemoautotrophic [30]. Previously studies have shown that addition of organic acids can improve the growth of *N. maritimus* [27], furthermore, they demonstrate that the metabolism of some Thaumarchaeota keeps activated when nitrification inhibitor was added [79].

Two of the genomes (ThauP41 and ThauR71) suggest participation in nitrification; however, the most likely reason that AOA genes were not detected in ThauP25 is that this genome is not complete. The gene *nirK* was detected in all three genomes, indicating participation in the production of the greenhouse gas nitrous oxide, which was demonstrated by heterologous expression of *nirK* from *Nitrososphaera viennensis* [80]*.* The presence of genes encoding coenzyme F420 are widespread in Thaumarchaeota genomes [9, 81]. This coenzyme has been detected in high amount in a proteomic study [82], suggesting an important role in its metabolism. The most probable hypothesis for this coenzyme is a protective role against reactive nitrogen species like observed in mycobacteria [83].

## Conclusions

Thaumarchaeota is the most abundant archaeal phylum in the Amazon River and Amazon River plume. Both areas are dominated by taxa closely related to *Candidatus* Nitrosotenuis uzonensis. The relative abundance of these Thaumarchaeota is associated with neutral to acidic pH and higher concentrations of nitrate, nitrite, and ammonia. The three genomes obtained in this study provide evidence of a typical Thaumarchaeota metabolism, with energy production by nitrification and carbon assimilation through the HP/HB cycle, suggesting that they are autotrophs. The presence of genes related to the TCA cycle and glycolysis suggest mixotrophic metabolism. In addition, these genomes contain genes involved in the production of nitrous oxide, and vitamins. Similarities between the genomes of Thaumarchaeota from the Amazon River and its plume suggest that all three organisms are present in both habitats, with the Amazon River representing a potential source of Thaumarchaeota in this habitat, where these organisms are of paramount importance.

## Methods

### Sampling and DNA extraction

We collected and analyzed 12 water samples from the Amazon River and its plume as described by Silva et al. 2017 [57]. Microbial DNA from samples preserved in SET buffer (20% sucrose, 50 mM EDTA, 0.5 mM Tris HCl) was extracted using the NucleoSpin Tissue kit (Macherey-Nagel, Dueren, Germany) with the following modifications for DNA extraction from Sterivex™ filters [84]: after incubation with lysozyme (final concentration 0.1%) at 37 °C for 60 min, 20% SDS (final concentration 1%) with proteinase K was added to the samples at 37 °C for 30 min, followed by washing with chloroform:isoamyl alcohol (24:1) solution. The extracted DNA was analyzed by agarose gel electrophoresis (1% agarose in 1× Tris-borate-EDTA) and then quantified spectrophotometrically with a NanoDrop ND-1000 (Thermo Scientific, DE, USA) and enzymatically with a Qubit High Sensitivity Fluorometer (Life Technologies). The DNA was amplified with the GenomiPhi V3 Kit (GE Healthcare) according to the manufacturer’s instructions but with the amplification time increased to 16 h or 18 h [85].

### Illumina library construction and sequencing

DNA libraries were prepared using the Nextera XT Sample Preparation Kit (Illumina). Library size distribution was determined using the Agilent 2100 Bioanalyzer and High Sensitivity DNA Kit (Agilent) and quantified using the Applied Biosystems 7500 Real Time PCR System and KAPA Library Quantification Kit (Kapa Biosystems). The PhiX sequencing control v3 (Illumina) was added at 1%, and paired-end sequencing (2 × 250 bp) was performed on a MiSeq System (Illumina).

### Metagenomic analysis, binning, and annotation

Reads were trimmed and quality checked using Sickle version 1.33 with default parameters. The reads of the five samples of Amazon River (Tapajós, Óbidos, North Macapá, South Macapá, and Belém) were combined and then assembled into scaffolds using IDBA-ud [86]. The same was proceed for the seven samples (St1, St3, St4, St6, St10, St11, and St15) of the Amazon River plume. All metagenome data from each sample were uploaded to ggKbase.

The microbial community classification was based on phylogenetic makeup among the genes that compose a specific contig. When more than 50% of the genes that make up a contig belong to an organism in the NCBI database, taxonomic attribution is given. The taxonomy of sequences from the Amazon River and its plume was visualized using the “View project taxonomy” tool. All this information is available on the website (https://ggkbase-help.berkeley.edu/).

Genomes were binned using ggKbase binning tools (ggkbase.berkeley.edu) and manually curated to remove scaffolds with anomalous GC content, coverage, or taxonomic classification [87]. Genome completeness was estimated based on 38 archaeal single-copy genes, as described previously [88, 89]. Each assembled genome was uploaded to the Integrated Microbial Genomes (IMG) system at the US Department of Energy’s Joint Genome Institute for annotation [90, 91].

Protein-coding genes of ThauP25, ThauP41, ThauR71, and N4 were assigned to COG & KOG functional category [55] by comparison to COG PSSMs using RPS-BLAST with E-value cutoff of 1e-2, and minimum alignment length of 70% of the consensus sequence length with the top hit [91].

Metabolic reconstruction and pathway mapping of each assembled genome was completed using the KEGG Automatic Annotation Server [92].

Catalytic and carbohydrate-binding modules of enzymes that degrade, modify, or create glycosidic bonds were identified by searching against the Carbohydrate-Active enZYmes database (http://www.cazy.org/) [93] using Diamond version 0.9.22 [94] with E-value cutoff of <1e-102. These enzymes and associated modules include glycoside hydrolases (GHs), enzymes with auxiliary activities (AAs), carbohydrate esterases (CEs), glycosyl transferases (GTs), and carbohydrate-binding modules (CBMs).

We construct a Venn diagram using the gplots package in R software [95] to identify unique and shared genes, utilizing protein-coding genes with predicted function of *Candidatus* Nitrosopelagicus brevis bin25 (ThauP25; IMG Genome ID 2781125713), *Candidatus* Nitrosotenuis sp*.* bin41 (ThauP41; IMG Genome ID 2781125712), *Candidatus* Nitrosotenuis sp. bin71 (ThauR71; IMG Genome ID 2778260922), and *Candidatus* Nitrosotenuis uzonensis N4 (N4; IMG Genome ID 2619619229). We compared the genomes acquired in this study (ThauP25, ThauP41, and ThauR71) with the genome of *Candidatus* Nitrosotenuis uzonensis N4, isolated from geothermal environments [96].

### Phylogenetic analysis

To construct the phylogenetic tree, sequences of the concatenated ribosomal genes S11, S7, S2, L21, L11, and L3 were aligned with MAFFT version 7.388 [97], using default parameters, and then manually edited to delete the extra end of the long sequences. The phylogenetic tree was constructed in PhyML version 20,131,022 [98] with the BLOSUM62 matrix and 1000 bootstrap replications. The Average Nucleotide Identity (ANI) was calculated using a Pairwise ANI tools available on IMG [90, 99]. The GenBank accession numbers of these sequences are available in Additional file 1: Dataset S1.

## Supplementary information

**Additional file 1:** **Dataset S1.** provides GenBank accession numbers and ribosomal gene sequences for the genomes used to construct the phylogenetic tree.

**Additional file 2:** **Table S1.** lists the protein families of the Carbohydrate-Active enZYmes database detected in the genomes of ThauP25, ThauP41, and ThauR71.

## Data Availability

Genomes described in this article are available on the Integrated Microbial Genomes system (Accession numbers: ThauP25; IMG Genome ID 2781125713, ThauP41; IMG Genome ID 2781125712, ThauR71; IMG Genome ID 2778260922). Accession numbers of sequences used to construct the phylogenetic tree are available in the Supplemental Material Dataset S1.

## Abbreviations

ANI: Average Nucleotide Identity
AOA: Ammonia-oxidizing archaea
CDS: Coding sequence
CE: Carbohydrate esterase
COG: Clusters of orthologous groups
DNA: Deoxyribonucleic acid
GH: Glycoside hydrolase
GT: Glycosyl transferase
HP/HB: Hydroxypropionate/Hydroxybutyrate
PE: Paired-end sequencing
RNA: Ribonucleic acid
rRNA: Ribosomal RNA
TCA: Tricarboxylic acid cycle tRNA Transfer RNA
