# Supplementary material for: Genome-resolved metagenomics analysis provides insights into the ecological role of Thaumarchaeota in the Amazon River and its plume
Source: BMC Microbiol. 2020 Jan 15;20:13. doi: 10.1186/s12866-020-1698-x (PMC6964070; doi:10.1186/s12866-020-1698-x)
Supplement: Supplementary file 2 — Additional file 2: Table S1. lists the protein families of the Carbohydrate-Active enZYmes database detected in the genomes of ThauP25, ThauP41, and ThauR71. [file 12866_2020_1698_MOESM2_ESM.docx]

|  | ThauP25 | ThauP41 | ThauR71 |
| --- | --- | --- | --- |
| AA1 | 15 | ND | ND |
| GH1 | 5 | ND | ND |
| GT1 | 19 | 25 | 38 |
| GT2 | 123 | 63 | 52 |
| GT66 | ND | 20 | 46 |
| GT7 | ND | ND | 2 |
| GT90 | ND | ND | 2 |
| Total | 162 | 108 | 140 |

# Table S1 Number of genes belonging to protein families of the Carbohydrate-Active enZYmes Database detected in three Thaumarchaeota genomes obtained from metagenomic data of the Amazon River and its plume (E-value < 1e-102).

# S

# Abbreviations: AA, auxiliary activity; CE, carbohydrate esterase; GH, glycoside hydrolase; GT, glycosyl transferase; ND, not detected.

# ­
